# Phasing of single DNA molecules by massively parallel barcoding

**DOI:** 10.1038/ncomms8173

**Published:** 2015-06-09

**Authors:** Erik Borgström, David Redin, Sverker Lundin, Emelie Berglund, Anders F. Andersson, Afshin Ahmadian

**Affiliations:** 1 Science for Life Laboratory, Division of Gene Technology, School of Biotechnology, Royal Institute of Technology (KTH), SE-171 65 Solna, Sweden

## Abstract

High-throughput sequencing platforms mainly produce short-read data, resulting in a loss of phasing information for many of the genetic variants analysed. For certain applications, it is vital to know which variant alleles are connected to each individual DNA molecule. Here we demonstrate a method for massively parallel barcoding and phasing of single DNA molecules. First, a primer library with millions of uniquely barcoded beads is generated. When compartmentalized with single DNA molecules, the beads can be used to amplify and tag any target sequences of interest, enabling coupling of the biological information from multiple loci. We apply the assay to bacterial 16S sequencing and up to 94% of the hypothesized phasing events are shown to originate from single molecules. The method enables use of widely available short-read-sequencing platforms to study long single molecules within a complex sample, without losing phase information.

## Abstract (web summary)

DNA phasing information — the determination of which specific sequences belong to the same DNA molecule—is not easily obtained from sequencing applications that rely on short reads. Here the authors develop a phasing method based on massively parallel barcoding of single DNA molecules.

## Graphical abstract

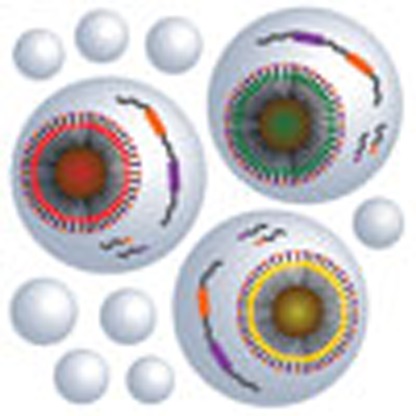

The vast majority of massively parallel sequencing data currently produced is of short-read length. Due to the short reads, such data sets typically lack phase information, that is, the physical connection of sequence variants is lost. Reliable phase information is important for a range of applications, for example, for resolving structural variants^1^ and assigning variants to alleles (haplotyping)^2^ in human genetic studies, for assessing alternative splicing in gene expression analysis^3^ and for linking functional genes to taxonomic groups in microbial community analysis^4^. Computational phasing is used to, for example, improve single-nucleotide polymorphism calling in population-based genotyping data and for reconstructing full-length 16S ribosomal RNA (rRNA) genes in metagenomic data^5^ but these techniques suffer from high error rates and are not capable of resolving rare or novel variants^6^. The development of sequencing technologies with long read length^7^,^8^ promises an alternative solution, but until the throughput and quality of such technologies have increased significantly, other approaches are needed.

Phase information can theoretically be reconstructed from short-read data if every read can be traced back to a single molecule. Several such methodologies for increasing the apparent read length of massively parallel sequencers has been demonstrated recently^9^,^10^,^11^,^12^. In the latest approach, pools of genomic fragments were combined in wells with specific barcodes, enabling well-stratified assembly and generating long contigs. Although proven effective, the throughput of the method is limited since it relies on physical separation of DNA in wells and use of predefined barcodes. The use of emulsion droplets instead of well plates would increase the reaction throughput by orders of magnitude. Emulsion droplets are also quick to generate and easy to scale. Yet associating each emulsion droplet with a unique barcode is an issue that remains to be solved. Recently, a procedure for labelling single DNA molecules with distinct, randomly generated barcode sequences was developed^13^,^14^. However, this approach does not solve the problem of introducing an identical barcode at multiple loci in the same DNA molecule.

We aim to address these issues by presenting a novel technique that in a high-throughput manner separates single molecules into individual compartments and uniquely barcodes the DNA molecule present in each compartment. The tagged molecules are then sequenced and the molecular origin of each read is traced back, enabling phasing of variants and long apparent read lengths. To achieve unique barcoding of single DNA molecules, a two-step emulsion compartmentalization approach was devised using beads as solid support.

Figure 1 describes the main conceptual steps of the method. The first emulsion reaction (steps I–IV) generates a primer library containing millions of clonally amplified barcode primers, while the second emulsion reaction (steps V–VIII) links sequence data from multiple loci of a single genomic fragment to a unique barcode. In step I, a water-in-oil emulsion is made, creating millions of discrete compartments where reactions take place. By dilution of a barcoding oligonucleotide, active compartments are generated that contain all of the following components; a single primer-coated bead, a single barcoding oligonucleotide and customizable amplification primers (step II). The barcoding oligonucleotide features a stretch of 15–20 degenerate bases surrounded by general handles. The degenerate stretch will be unique for any one molecule and functions as the barcode. The handle sequences enable connection of the barcode sequence and the custom target-specific primer sequences to the bead through PCR amplification (step III). Finally, emulsion breakage and enrichment of DNA-covered beads is performed, yielding a uniquely barcoded primer library on solid support (step IV). The barcoded primer library is then added to a diluted sample of DNA molecules and compartmentalized a second time (step V). Active compartments here contain a uniquely barcoded bead, a second set of amplification primers and a single genomic fragment (step VI). Amplification is performed within these compartments to exclusively couple the bead-bound barcodes to amplicons of the single genomic fragment (step VII). Following PCR, target amplicons are enriched and prepared for sequencing (step VIII). See Supplementary Figs 1 and 2 for a more detailed schematic view of the method.

## Results

### Validation of bead barcode monoclonality

**Figure 2:**
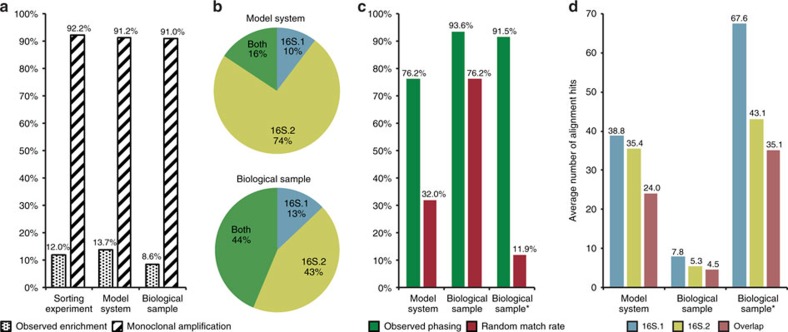
Results illustrated. Monoclonality and phasing results for the sorting experiment, the model system, the biological sample and the biological sample after removal of the most abundant species (biological sample*). (**a**) The rate of enriched beads and monoclonal amplification observed for each sample (see Supplementary Fig. 3 for corresponding theoretical values). (**b**) Proportion of amplicon-carrying beads displaying the 16S.1, 16S.2 or both the targeted regions. (**c**) The observed rate of matching BLAST-based classifications of bacterial origins before (green) and after (red) random shuffling of the data set, for all data sets. (**d**) The average number of alignment hits for the two target regions and the overlap between them.

To validate the method and demonstrate its applicability, a number of key experiments were constructed. Initially, a technical validation of the method was carried out by sorting individual barcoded beads from the first emulsion reaction into wells. The barcodes present in each well were then amplified and sequenced. Sequenced products (92.2%) displayed monoclonality (Fig. 2a; Supplementary Note 1; Supplementary Table 1), while the remaining wells predominantly exhibited two different barcodes. These results closely model the Poisson distribution both in terms of monoclonality and enrichment (Supplementary Note 1; Supplementary Fig. 3), confirming that the droplets are stable and the beads uniquely tagged, making them suitable for phasing of single DNA molecules.

### Model system for phasing of single molecules

A model system was designed to investigate tracking of separate amplicons to a single fragment of origin. Two variable 16S rRNA gene regions (hereafter referred to as 16S.1 and 16S.2; Supplementary Fig. 4) were amplified from a mixture of four known bacterial genomes. The experiment generated 3,029 clusters of read pairs with unique barcode sequences, each representing an amplicon-carrying bead (henceforth referred to as barcode clusters or clusters; Supplementary Note 2). Out of these, 91.2% displayed monoclonal amplification of the targeted region(s). In all, 10.3 and 74.0% of the total amount of barcode clusters had amplified 16S.1 and 16S.2, respectively, while both amplicons could be identified for 15.7% of the clusters (Fig. 2b). Clusters featuring both targets can stem from two separate molecules even though both amplicons have been monoclonally amplified. Therefore, the rate of successful single-molecule phasing was evaluated by classifying and matching the bacterial origin of each amplicon within each cluster. Bacterial origins were identified by alignment of all amplicon sequences against a database of known bacteria. In 76.4% of the barcode clusters, the 16S.1 and 16S.2 amplicon sequences were of identical bacterial origin. To validate this phasing rate, amplicon sequences were also matched randomly between different clusters. This random match rate was 32.0%, somewhat higher than 25% expected from an even distribution of four genomes. Further analysis shows that the observed difference is supported by a skewed representation of the four genomes observed in our sample. The phasing rate was corrected using the random match rate, providing a minimum rate of 65.8% of the clusters displaying phased data from single molecules. For detailed results, see Supplementary Note 2; Supplementary Fig. 5.

### Phasing of single molecules from a biological sample

The same two targeted regions were amplified from a complex biological sample to investigate the potential of the protocol in a real case scenario. This experiment was performed at a larger scale and generated 66,000 barcode clusters (Supplementary Note 3). A high rate of monoclonal amplification of the targeted regions (91.0%; Fig. 2a) was observed, close to the model system and the theoretically expected value. Compared with the model system, a considerably larger proportion of beads had successful amplification of both target regions (43.7%). A simulation of different sequencing depths indicate that this difference can be explained by an increase in sequencing depth for the biological sample, resulting in more clusters with sufficient reads from each amplicon to pass the defined filtering steps (Supplementary Note 4; Supplementary Fig. 6). As observed in the model system, the 16S.1 amplicon was less prevalent than the 16S.2 amplicon (Fig. 2b; Supplementary Note 4). The phasing was determined as successful for 93.6% of the barcode clusters (as previously described). However, the random match rate was also high (76.2%), indicating that the biological sample contained one dominating species. This was investigated further (Supplementary Note 3; Supplementary Fig. 7) and after removing sequences aligning to the most abundant species from the analysis, the random match rate dropped to 11.9%, while the rate of phased barcode clusters remained high at 91.5% (Fig. 2c). The phasing rate was adjusted as described earlier, giving a rate of 90.3% of the clusters displaying truly phased single-molecule data.

To investigate the benefit of phasing on a biological study, we counted and compared alignment hits for both of the amplicons. The overlap between the two lists of alignment hits was compared with the length of each individual hit list to see whether phasing made classification less ambiguous (Fig. 2d). We observed that with phase information the list of potential species were shorter than when individual amplicons were analysed. This reduction was 32.2 and 15.5% for the model system and biological system, respectively (Supplementary Note 4).

## Discussion

In this study, we have presented a method that in a high-throughput manner enables unique barcoding, monoclonal amplification and phasing of amplicons from single DNA molecules in discrete compartments. A primer library of nearly 2 million uniquely barcoded beads was generated in the first emulsion reaction. Sorting and sequencing of individual beads validated the monoclonal amplification within emulsion droplets. Around 91% of the barcode clusters in each data set were monoclonal for the targeted amplicon(s). We then progressed to validate single-molecule resolution by investigating phasing of multiple amplicons. Although we observed an uneven representation of the species in the sample, the phase information was maintained in up to 93.6% of the monoclonal barcode clusters, confirming that the vast majority of the data is generated from successfully barcoded single DNA molecules.

Emulsions are easily generated and the price of traditional short-read high-throughput sequencing drops continuously. The method presented here is, therefore, very flexible in terms of scale compared with the long-read-sequencing approaches available to date. The base calling quality is also higher than what can be achieved by long read sequencers. Furthermore, the data displays true single-molecule resolution as opposed to the limiting dilution and tagging of molecule groups used by previous phasing strategies. Without this one-to-one relationship between barcode and DNA molecule there is a risk of clustering short reads from several DNA fragments to one consensus. Another advantage of single-molecule resolution is that the assay becomes independent of sample complexity. As a result, our approach enables analysis of highly similar sequences or close to identical targeted regions such as rRNA genes from different bacterial species.

The method is relevant for a wide range of applications where phasing, or coupling of biological information, is of interest. The genomic distance separating phased amplicons is dependent only on the size of the genomic fragments added to the second emulsion reaction. Depending on the application, the maximum distance that can be phased will, therefore, be determined by the quality of the input material and to what extent the molecules enter the compartments. Considering fragment sizes of routinely extracted genomic DNA, the maximum phasing distance, for single-molecule fragments, is expected to be in the range of 40 kb. For targeted approaches where an enrichment of the sample may be required, the ability to generate long input sequences by long-range PCR will determine this fragment size. Long-range PCR is a technique routinely used to amplify large genomic regions and commercial kits are available for amplicons of 5–30 kb in size. The feasibility of such an approach has been illustrated in a separate experiment by performing amplification of the full targeted region before applying our method to the sample material (Supplementary Note 6). Our method could, thus, potentially be used for characterization of polymorphisms within HLA genes, where the ability to resolve haplotype information would not only improve the accuracy of HLA typing but also enable researchers to probe the relationship between genetic variation and disease susceptibility^1^.

It is worth noting that the method does not produce a continuous phased sequence but rather a phased coupling of target regions. Increasing the number of amplicons would improve the coverage of the target region. Developing a target-specific approach that is highly multiplex is inherently limited by cross-talk between the primers. To circumvent this, our approach needs to be adapted to highly multiplex amplification techniques such as golden gate^15^ or trinucleotide threading^16^. As these assays employ universal handles, they could easily be implemented in the here presented amplicon barcoding workflow. However, both assays would require a pair of target-specific probes for each region. To further increase coverage and acquire a close to continuous sequence, we envision an assay that increases the multiplicity of phased regions without the use of target-specific probes. With minor adjustments to the current assay, this could be achieved by means of random amplification. Combining our approach with multiple displacement amplification reactions in our emulsion droplets should drastically improve the coverage or proportion of phased sequence data obtained from each genomic fragment. As emulsions are easy to scale, such an approach could be used to phase the entire human genome in one experiment. By enabling complete phasing of large genomic fragments, such a technology would also substantially reduce the bioinformatical load of genome assembly for *de novo* sequencing.

By compartmentalization of single cells with the bead-bound primer library, our method for tagging the contents in individual droplets could be applied for analysis of single cells rather than single DNA molecules. This in combination with substitution of the target-specific primers for a poly-T handle to capture messenger RNA would enable high-throughput single-cell transcriptome profiling. Independent molecular profiling of each cell within a sample would for instance enable massively parallel single-cell sequencing of complex microbial communities, and could drastically improve on currently available techniques for analysing circulating tumour cells in heterogeneous samples.

## Methods

### Bead-bound primer library generation

A barcoding oligonucleotide (Supplementary Tables 2a,b) containing 15 degenerated bases (20 bases for sorting experiment) surrounded by general handle sequences was introduced into emulsion droplets with beads, target-specific amplification primer(s) and other emulsion reagents as specified by the 454 Sequencing emPCR Method Manual Lib-L SV (Roche, Penzberg, Upper Bavaria, Germany). The only exception in terms of reagents used in the protocol was the amplification primer(s), which were designed in-house and purchased from Eurofins MWG (Ebersberg, Germany). These customized target-specific amplification primer(s) were added to the ‘Live Amplification Mix' instead of the ‘Amplification Primer' accompanying the 454 Lib-L SV Kit (Roche). The number of target-specific primers is dependent on experimental design, and consequently this detail is different for the sorting and the phasing experiments (Supplementary Tables 2a,b). The final concentration for each target-specific primer used in an emulsion reaction was kept at 1 μM. The aqueous phase (denoted ‘Live Amplification Mix' in the 454 method manual) was prepared with ∼0.1 barcode oligonucleotide molecules per bead (hereafter referred to as copy per bead or c.p.b.). The use of 0.1 c.p.b. is equivalent to 240,000 oligonucleotide molecules per 2.4 million beads (standard bead input per emulsion reaction in the 454 Lib-L SV kit). PCR cycling was carried out in a Mastercycler Pro S (Eppendorf, Hamburg, Germany) instrument, in accordance with the emPCR method manual previously referenced.

A single emulsion reaction was generated with one target-specific primer ‘FACS Amplification' Supplementary Table 2a) for the sorting experiment to validate monoclonal amplification. For the model system and biological system, two and ten emulsion reactions (Supplementary Fig. 1), respectively, were generated in parallel using two target-specific forward primers (16S.1.Fwd and 16S.2.Fwd; Supplementary Table 2b).

Following the emPCR, emulsion breakage and enrichment were carried out in accordance with 454 specifications, with the exception of using 6 μM of custom-enrichment primers (Supplementary Tables 2a,b) instead of the ‘Enrichment Primer' accompanying the 454 Lib-L SV Kit (Roche). After enrichment and washing, the beads were resuspended in 200 μl Annealing Buffer XT (Roche) and counted by withdrawal of a 3-μl aliquot, using a Coulter Counter (Beckman, Brea, CA, USA). The output of enriched beads is expected to be roughly equivalent to the calculated input of barcoding oligonucleotides. Thus, with an input 0.1 c.p.b. barcoding oligonucleotides, the enrichment output is expected to be ∼10% of the number of beads used in the emulsion.

When the beads are enriched and counted, the primer library is ready to be incorporated into the second emulsion reaction for phasing of DNA fragments, which is executed in different ways depending on experimental design (see following sections below). Multiple emulsion reactions performed for the model system and biological systems were broken and enriched in parallel. The enriched and counted beads were then pooled to form a single primer library featuring 1.8 million clonally amplified barcoded beads covered with forward primers for both 16S.1 and 16S.2 regions (for the biological system).

### Sorting individual barcoded beads

A single compartmentalization reaction was prepared as described in the previous section. Following enrichment, beads were individually sorted into 96-well plates by means of fluorescence activated cell sorting (FACS). A 100-μl aliquot of the enriched beads was mixed with 20 μM of a fluorescently labelled primer (Supplementary Table 2a), incubated at 55 °C for 5 min, and then put on ice. The beads were then washed with 1 ml Elution Buffer (Qiagen, Hilden, Germany). The beads were sorted to individual PCR plate wells, by considering both the bead size and fluorescence using a BD Influx instrument (Becton Dickinson, Franklin Lakes, New Jersey, USA).

To tag each sorted bead with a well-specific id-tag, PCR was carried out in a total volume of 50 μl per well. Each PCR reaction contained 1 × PCR Buffer-Mg (Invitrogen, Carlsbad, CA, USA), 200 μM dNTPs (Invitrogen), 1.5 mM MgCl_2_ (Invitrogen), 1 U Platinum Taq DNA Polymerase (Invitrogen), 100 nM 454 bead handle (Supplementary Table 2b) and 100 nM of a well-specific id-tag^17^. The reaction was cycled in a Mastercycler Pro S (Eppendorf) under the following conditions: 2 min of initial denaturation at 94 °C, followed by 25 cycles of (i) 30 s denaturation at 94 °C, (ii) 30 s annealing at 52 °C and (iii) 30 s extension at 72 °C. After cycling, the protocol ended with 5 min extension at 72 °C.

Library amplification was performed separately on 1 μl from each of the PCR reactions, in a total volume of 50 μl containing 1 × PCR buffer-Mg (Invitrogen), 200 μM dNTPs (Invitrogen), 1.5 mM MgCl_2_ (Invitrogen), 1 U Platinum Taq DNA Polymerase (Invitrogen), 500 nM PCR Primer PE 1.0 (Illumina, San Diego, CA, USA), 100 nM primer InPe 2.0 FACS (Supplementary Table 2a) and 500 nM of Indexing primer (Illumina). The PCR conditions were identical to those described in the previous paragraph.

One μl of each library was then electrophoresed with a LabChip GX DNA High Sensitivity kit (Caliper, Hopkinton, MA, USA) according to the manufacturer's instructions. Twenty μg of Proteinase K (Qiagen) was added to each sample well and incubated for 10 min at 25 °C, and then inactivated at 60 °C for 20 min. This was followed by pooling 5 μl of each well that contained a successfully amplified product (as determined by electrophoresis). A polyethylene glycol (PEG) precipitation on carboxylic acid solid support was performed to remove excess amplification primers as described previously^18^ using a Magnatrix 1,200 Biomagnetic Workstation (NorDiag ASA, Oslo, Norway) with 12.5% PEG in 1.5 M NaCl.

DNA concentration was measured using a Quant-iT dsDNA BR kit (Invitrogen) in accordance with the manufacturer's instructions. The sample was then diluted to 2 nM and sequenced using a MiSeq Personal Sequencer (Illumina). The data were analysed using in-house developed scripts (see below).

### Model system phasing of single molecules

A barcoded primer library was generated from two parallel emulsion reactions as previously described. These beads were used in a model system including four bacterial genomes. DNA extracted from the four bacterial genomes—*Paracoccus aminophilus* strain JCM 7686, *Bacillus amyloliquefaciens* strain FZB42, *Alteromenas macleodii strain* ATCC 27126 and *Escherichia coli* strain K-12/MG1655 (DNA purchased from LGC Standards, Teddington, Middlesex, UK) were added to the barcoded primer library. Target sequences were then captured by hybridization and extension by the following procedure:

The beads were suspended in 15 μl Annealing Buffer XT (Roche). Genomic DNA was diluted to a total of 0.1 c.p.b. (according to copy number of the 16S rRNA gene in the respective genomes), placed in a heating block at 95 °C for 2 min and then directly on ice for 2 min. The diluted DNA was then added to the beads and the mixture was placed back in the 95 °C heating block with shaking at 900 r.p.m. After 2 min at 95 °C, the temperature was slowly lowered to 80 °C, at which point Phusion HF 2 × Master Mix (Thermo Scientific) was added to a final concentration of 1 × . The temperature was then lowered to 58 °C with continued shaking at 900 r.p.m. After 10 min at 58 °C, the temperature was slowly increased to 72 °C for 20 min, after which the tube was put directly on ice for 2 min. After cooling, the tube was spun down at 10,000 r.p.m. for 20 s and the supernatant was discarded. The beads were then washed twice with 150 μl 1 × PCR Buffer-Mg (Invitrogen) cooled to 4 °C, by mixing the bead solution with a pipette and then spinning down before removing the supernatant (without disturbing the bead pellet). The beads were then suspended in 25 μl 1 × PCR Buffer-Mg (Invitrogen) and refrigerated until used in the second compartmentalization.

The beads were incorporated in a second emulsion reaction with a customized aqueous phase containing reverse primers 16S.1.Rev and 16S.2.Rev at 1 μM (Supplementary Table 2b). The emPCR preparation was carried out in accordance with the 454 Sequencing emPCR Method Manual Lib-L SV (Roche), excluding steps related to preparation and washing of fresh Capture Beads. With the forward primers attached to the beads and the reverse primers in solution, this emPCR generates 16S.1 and 16S.2 amplicons on the beads (Supplementary Fig. 1). The following amplification, emulsion breakage and enrichment steps were also carried out according to the method manual, with the exception of the enrichment primer used. An equimolar mixture (3 μM of each) of 16S.1.Enrich and 16S.2.Enrich primers were used to capture beads representing 16S.1 and/or 16S.2 amplicons. After this target-specific enrichment, the beads were counted using a Coulter Counter (Beckman).

### Biological sample phasing of single molecules

A barcoded primer library was generated from 10 parallel emulsion reactions as previously described. Ninety-five per cent of these beads were used for the biological sample, corresponding to ∼1.7 million beads. Bacterial DNA extracted from a complex biological sample (human faecal sample) was diluted and captured on the beads as previously described. The dilution was done according to copy number of the 16S rRNA gene, assuming an average of 4.2 copies per genome^19^ to get an input of 0.1 c.p.b. The diluted genomic material was coupled with the beads by means of hybridization and extension (as previously described for the model system). The following second emulsion reaction, emulsion breakage and enrichment steps were all carried out in the same way as previously described for the model system.

### Sequencing library preparation

Beads from the second compartmentalization were suspended in 25 μl Annealing Buffer XT (Roche) following emulsion breakage and enrichment. A linear PCR of the bead-bound amplicons was carried out with 1 × Phusion High Fidelity Master Mix (Thermo Scientific) and 1 μM Indexing primer (Illumina), using the following protocol: 98 °C for 1 min, followed by five cycles of 30 s at 98 °C, 2 min at 65 °C and 4 min at 72°C. After cycling, the protocol ended with extension at 72 °C for 5 min, before cooling down to 4 °C. The PCR tube was then spun down and the supernatant was transferred to a fresh PCR tube, to which PE PCR Primer 1.0 (Illumina) was added to a final concentration of 1 μM. The single stranded products were then made double stranded by annealing of the PE PCR Primer 1.0 (Illumina) at 65 °C for 5 min and extending at 72 °C for 20 min. The target amplicons were then purified by means of PEG precipitation as previously described. See Supplementary Fig. 2 for a schematic view of the described library preparation steps.

To amplify the double-stranded products, a PCR was performed with 1 × Phusion HF Master Mix, 500 nM PE PCR Primer 1.0 (Illumina) and 500 nM Indexing primer (Illumina). The following PCR protocol was used: 95 °C for 2 min, followed by cycling of 30 s at 95 °C, 2 min at 65 °C and 2 min at 72 °C, followed by 5 min at 72 °C. The number of PCR cycles was adjusted according to the quantity of input material (that is, the number of enriched beads). Four cycles were run for the biological sample, while six cycles were run for the model system. Carboxylic acid size exclusion was performed to remove excess primers and primer dimers (as previously described). The products were then verified by electrophoresis with LabChip GX DNA High Sensitivity kit (Caliper) using a 2100 Bioanalyzer instrument (Agilent, Santa Clara, CA, USA).

The DNA concentration was measured using Quant-iT dsDNA HS kit (Invitrogen), in accordance with the manufacturer's protocol, and sample was diluted to 2 nM. After quantification, libraries were sequenced using a HiSeq 2500 run in Rapid mode (2 × 100) and MiSeq (2 × 150) (Illumina) and the sequence data were analysed using in-house developed scripts (see below).

### Sorting experiment data analysis

The sequencing data from the sorting experiment was analysed by a custom python script. For each pool of amplified products, read pairs were initially sorted based on a barcode sequence identifying the well of the original FACS plate (Supplementary Fig. 8a). For each well, the most represented barcodes were chosen and all other barcode sequences were compared and grouped with these allowing for a predefined number of mismatches. This grouping procedure was repeated until no decrease in total number of barcodes was observed. The barcode distribution in each well was then evaluated manually to determine wherever the product should be classified as monoclonal or polyclonal (Supplementary Table 1; Supplementary Note 1). Both the grouping of similar barcodes and the sorting to predefined well barcodes allowed for two mismatches, while four mismatches were allowed in the handle sequence separating the two barcodes.

### Phasing experiments data analysis

To evaluate data resulting from model system and biological system, a custom data analysis pipeline was written as a set of python scripts. The scripts automate identification and clustering of barcode sequences, grouping of reads by these clusters, filtering of reads, clustering of reads with target-specific sequence content and classification of the bacterial origin of amplicon consensus sequences. Each step is described and motivated in more detail in Supplementary Note 5. All scripts used for the data analysis are available at https://github.com/elhb/SEAseq.

## Additional information

**How to cite this article:** Borgström, E. *et al*. Phasing of single DNA molecules by massively parallel barcoding. *Nat. Commun.* 6:7173 doi: 10.1038/ncomms8173 (2015).

**Accession codes:** All data from this study has been uploaded to the Sequence Read Archive (SRA), it has been assigned the accession number SRA248941.

## Supplementary Material

Supplementary Information Supplementary Figures 1-8, Supplementary Tables 1-2, Supplementary Notes 1-6

## Figures and Tables

**Figure 1:**
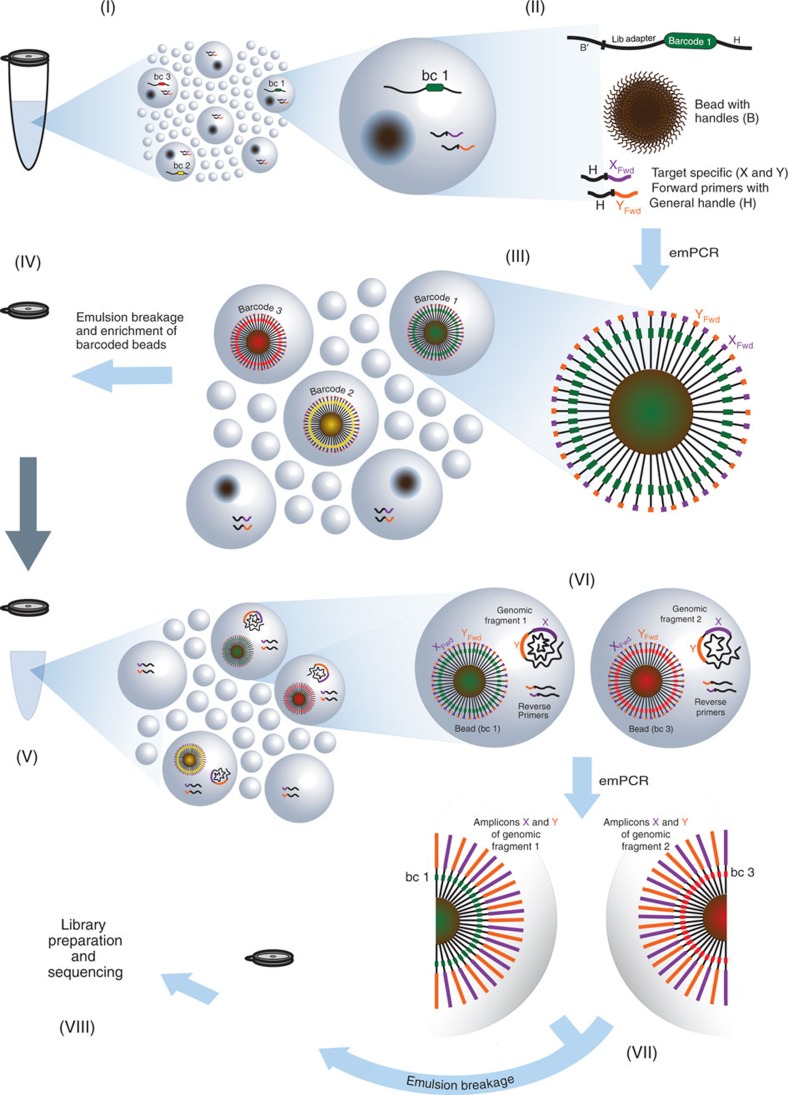
Method overview. (I) An emulsion is generated with reaction compartments and smaller stabilizing droplets. (II) An active compartment consists of amplification reagents and one molecule from a population of 4 (ref. 15) degenerate barcode oligonucleotides. By utilizing a subset of these molecules, we ensure unique barcoding of each bead. (III and IV) A library with millions of uniquely barcoded primer sets is generated and enriched. (V) The bead-bound primer library is mixed with a diluted sample of genomic fragments and then introduced into a second emulsion. (VI and VII) Each bead is paired with one genomic fragment and the target-specific amplicons are coupled with the barcode through amplification. (VIII) Barcoded amplicons are enriched and sequenced.
