# Supplementary material for: Phasing of single DNA molecules by massively parallel barcoding
Source: Nat Commun. 2015 Jun 9;6:7173. doi: 10.1038/ncomms8173 (PMC4468844; doi:10.1038/ncomms8173)
Supplement: Supplementary Information — Supplementary Figures 1-8, Supplementary Tables 1-2, Supplementary Notes 1-6 [file ncomms8173-s1.pdf]

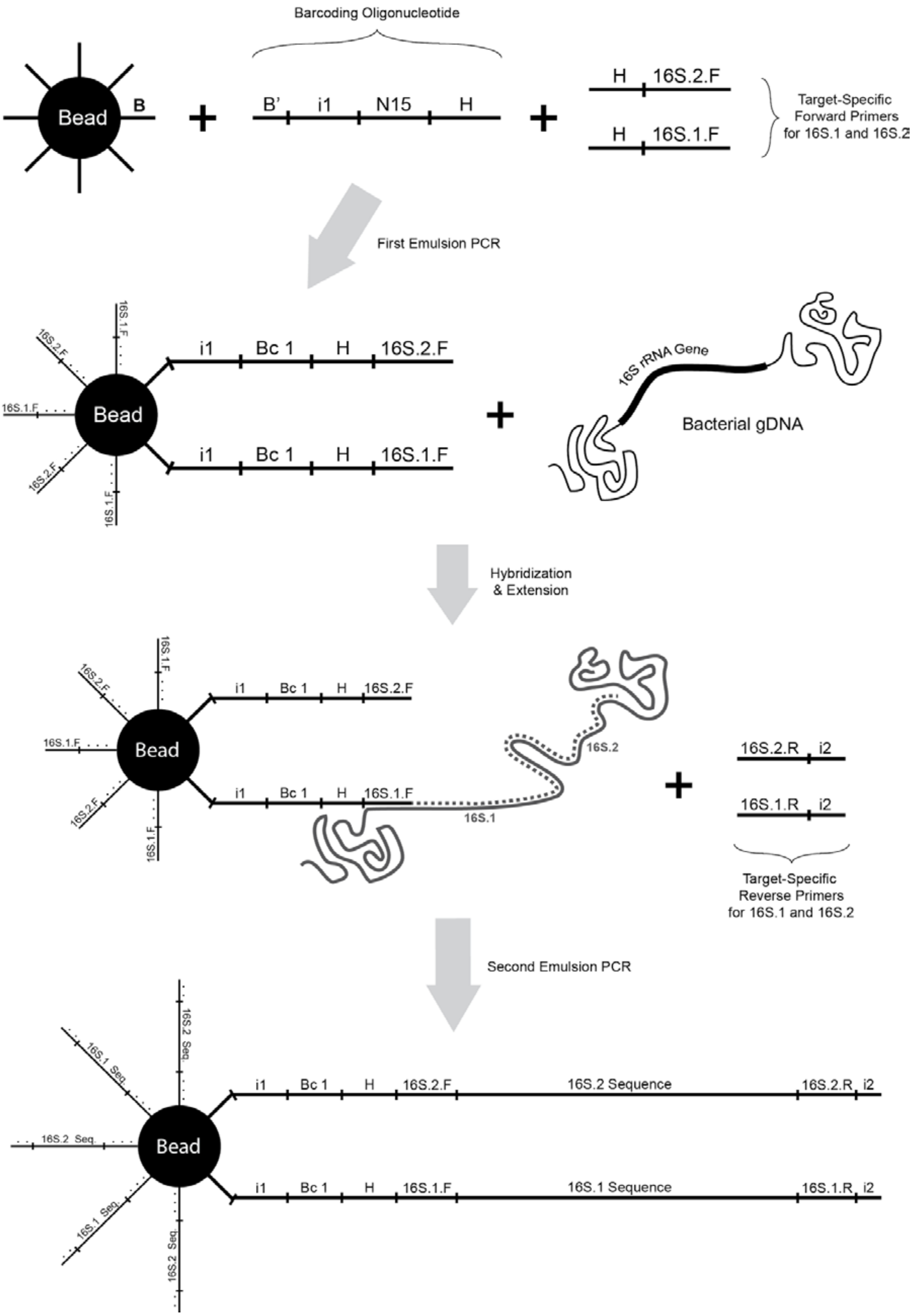

Supplementary Figure 1 | Schematic view of phasing approach. A sequence-based schematic view of the serial compartmentalization approach. First, barcoded primer sequences are attached to the bead surface in the first emulsion PCR. Second, the target-specific forward primers are used to capture a genomic target sequence. The bead-target complex is then amplified in a second emulsion PCR with target-specific reverse primers consisting of two parts, a target-specific portion and a general library adaptor (i2). The target-specific portion hybridizes to target downstream of the bead-bound primers and thereby marks the end of the desired amplicons.

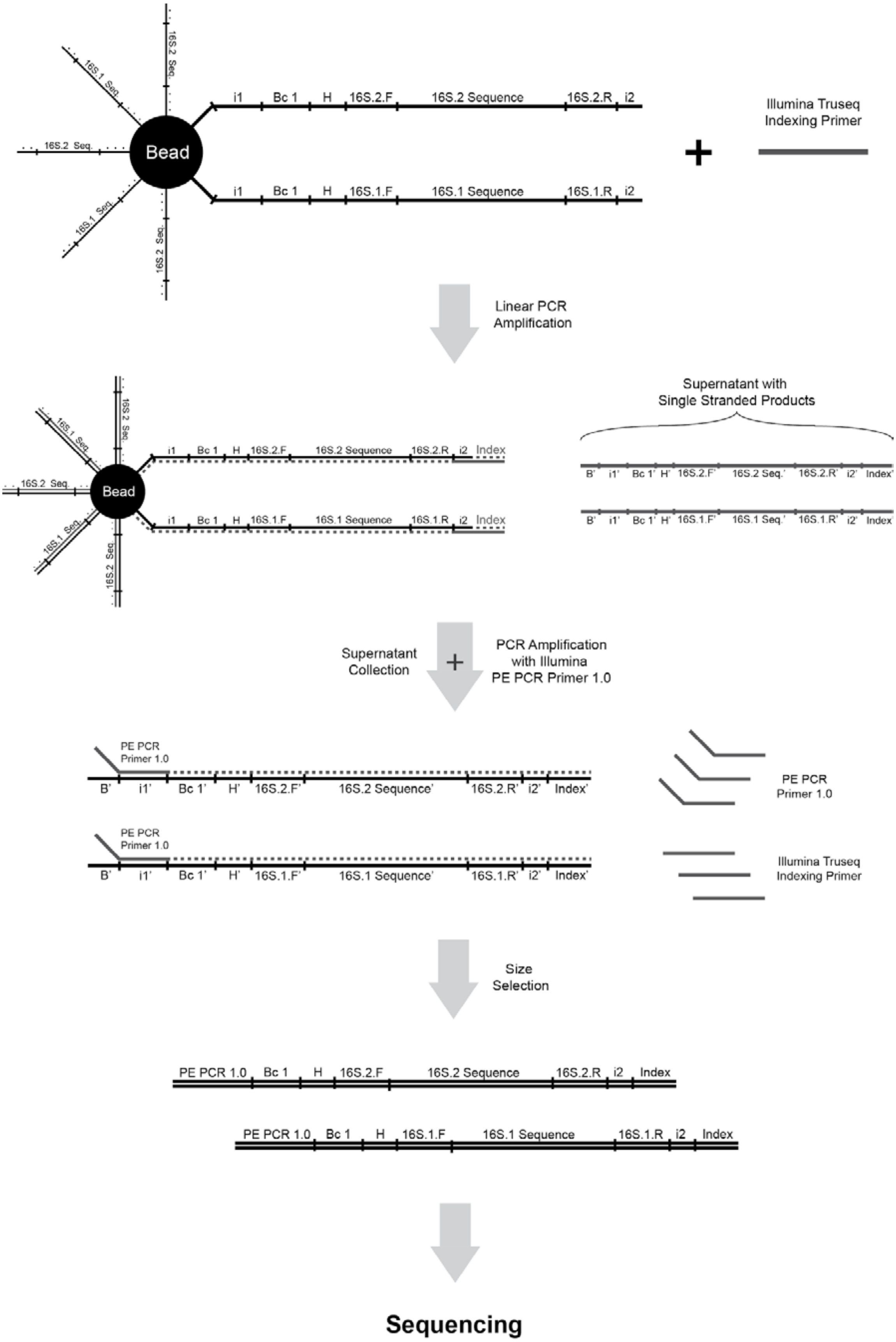

Supplementary Figure 2 | Schematic view of library preparation steps. A sequence-based schematic view of the library preparation steps following the second emulsion reaction and enrichment of target amplified beads. First, a linear PCR is performed with an Illumina Indexing primer to release the target amplicons from the bead surface. The resulting DNA in the supernatant is then amplified and made double stranded. Finally, short dsDNA bi-products and excess primers are removed before the sample is sent for sequencing.

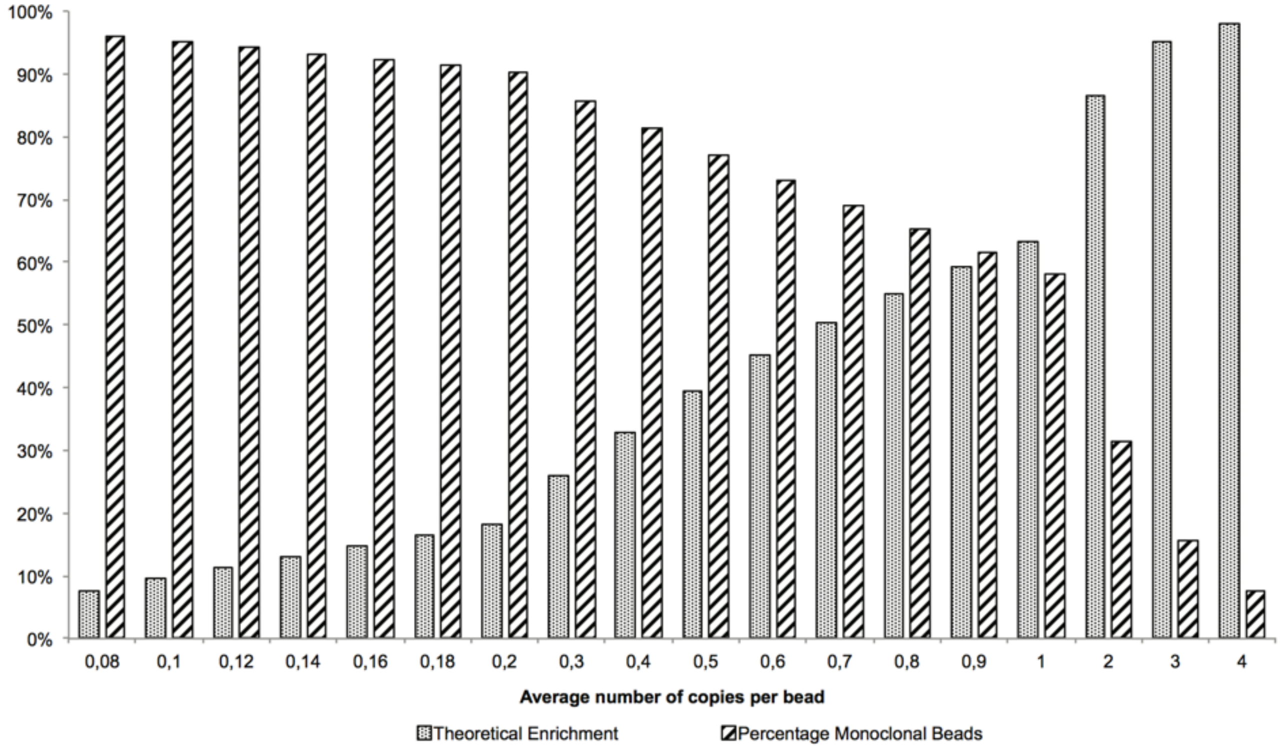

Average cpb	0,08	0,1	0,12	0,14	0,16	0,18	0,2	0,3	0,4	0,5	0,6	0,7	0,8	0,9	1	2	3	4
Theoretical Enrichment	7,7%	9,5%	11,3%	13,1%	14,8%	16,5%	18,1%	25,9%	33,0%	39,3%	45,1%	50,3%	55,1%	59,3%	63,2%	86,5%	95,0%	97,9%
Percentage Monoclonal Beads	96,1%	95,1%	94,1%	93,2%	92,2%	91,3%	90,3%	85,7%	81,3%	77,1%	73,0%	69,1%	65,3%	61,7%	58,2%	31,3%	15,7%	7,5%

Supplementary Figure 3 | Theoretical values according to the Poisson Distribution. The figure shows the expected enrichment and proportion of monoclonal beads according to the Poisson distribution for different values of average number of template molecules per bead.

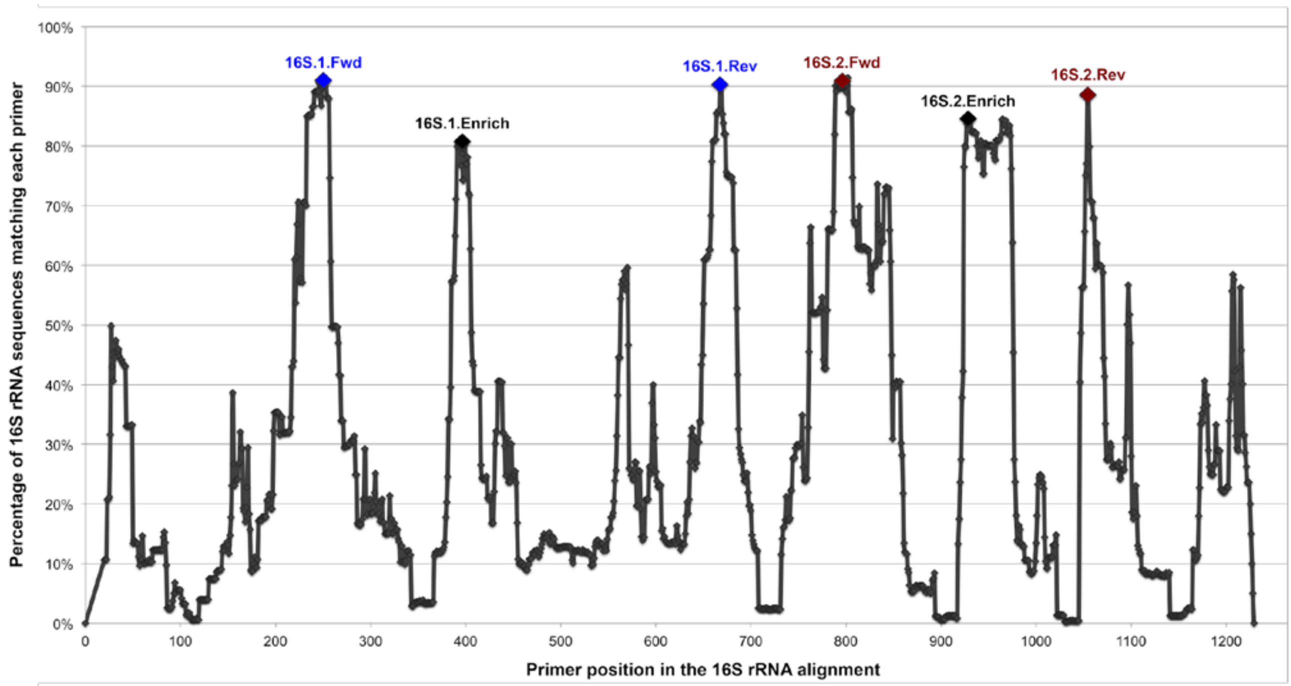

Supplementary Figure 4 | Primer design for bacterial 16S rRNA sequences. The figure shows a measure of sequence variability in bacterial 16S rRNA sequences. The vertical axis shows the percentage of species with sequence match to a potential 25 bp primer for each position in a multi alignment of all bacterial 16S sequences (obtained from the Ribosomal Database Project[1] (RDP) release 10.32). The position of each primer is relative to a trimmed alignment in which regions of very high variability have been removed (i.e. those which only exist in a handful of species out all known bacteria).

The location of the primer pairs used are marked in blue (for 16S.1 amplicon) and red (for 16S.2 amplicon). Although these regions do not overlap one can see that the two regions spans a majority of the 16S rRNA gene. Each primer was designed to hybridize to highly conserved regions within the 16S rRNA gene. Conversely, the majority of the amplified region between the forward and reverse primers are of high complexity, meaning that the sequence of different bacteria are highly variable within these regions. Exceptions to this are the positions of the enrichment primers (marked in black). The proportion of matching species for each specific 25-mer primer were obtained by alignment of the bacterial 16S rRNA genes using DegePrime[2]. The DegePrime software was run with a window size (primer length) set to 25 and maximum degeneracy set to 12. Primer sequences were then manually chosen to fulfill the following criteria; (i) maximum match rate, (ii) minimum degeneracy, (ii) GC content, (iii) hybridization temperature, and (iv) minimal tendency to form secondary structures to general handle sequences.

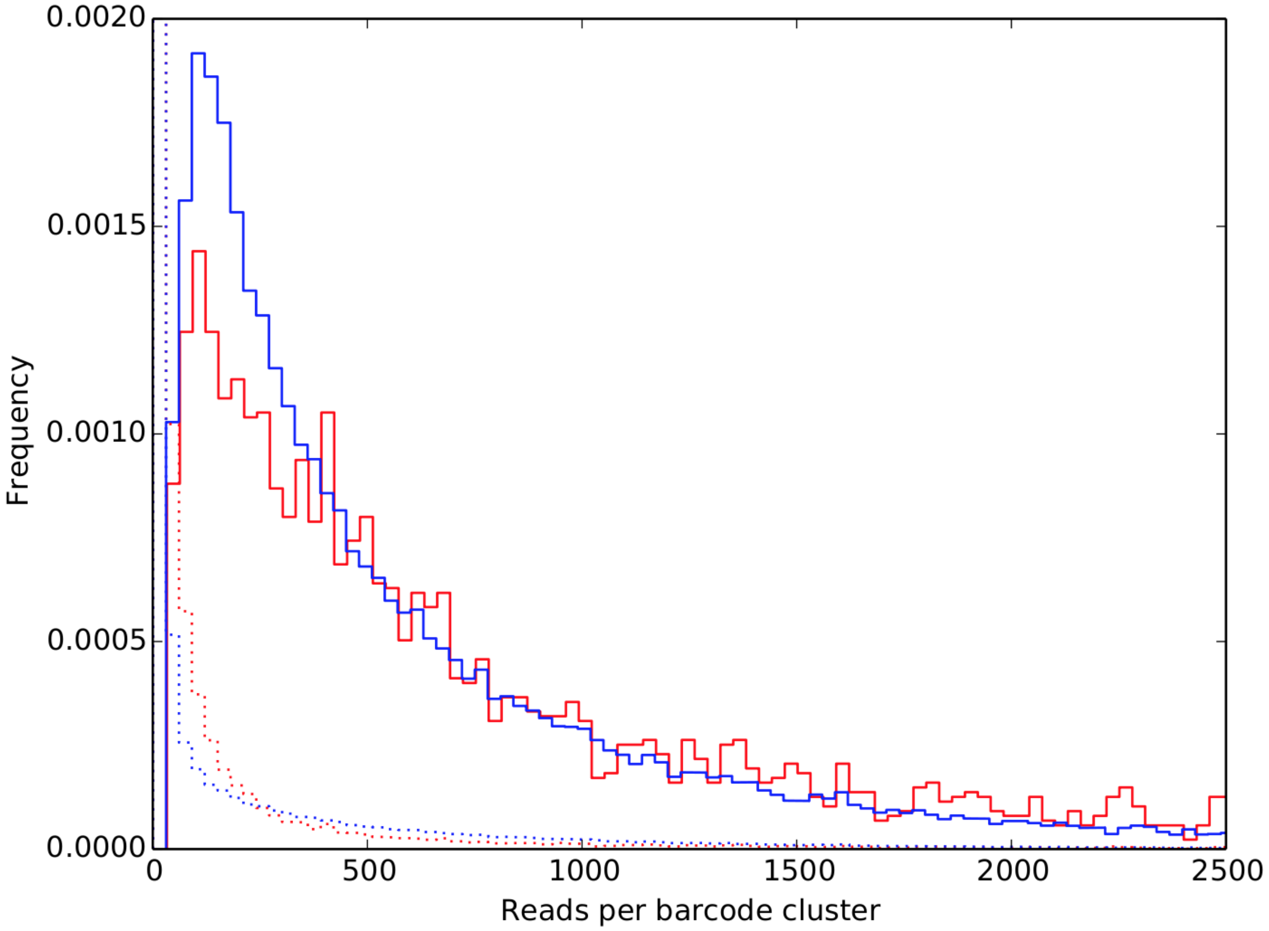

Supplementary Figure 5 | Histogram of reads per cluster for Model System and Biological Sample. The distribution of read counts per barcode cluster for model system cluster before (red dashed) and after (red solid) filtering as well as biological sample clusters before (blue dashed) and after filtering (blue solid). For each of these populations the total frequency of all clusters with more than 2500 reads were 0.0036, 0.0949, 0.0029, 0.0365 respectively. The histogram is binned into 30 read pairs bins.

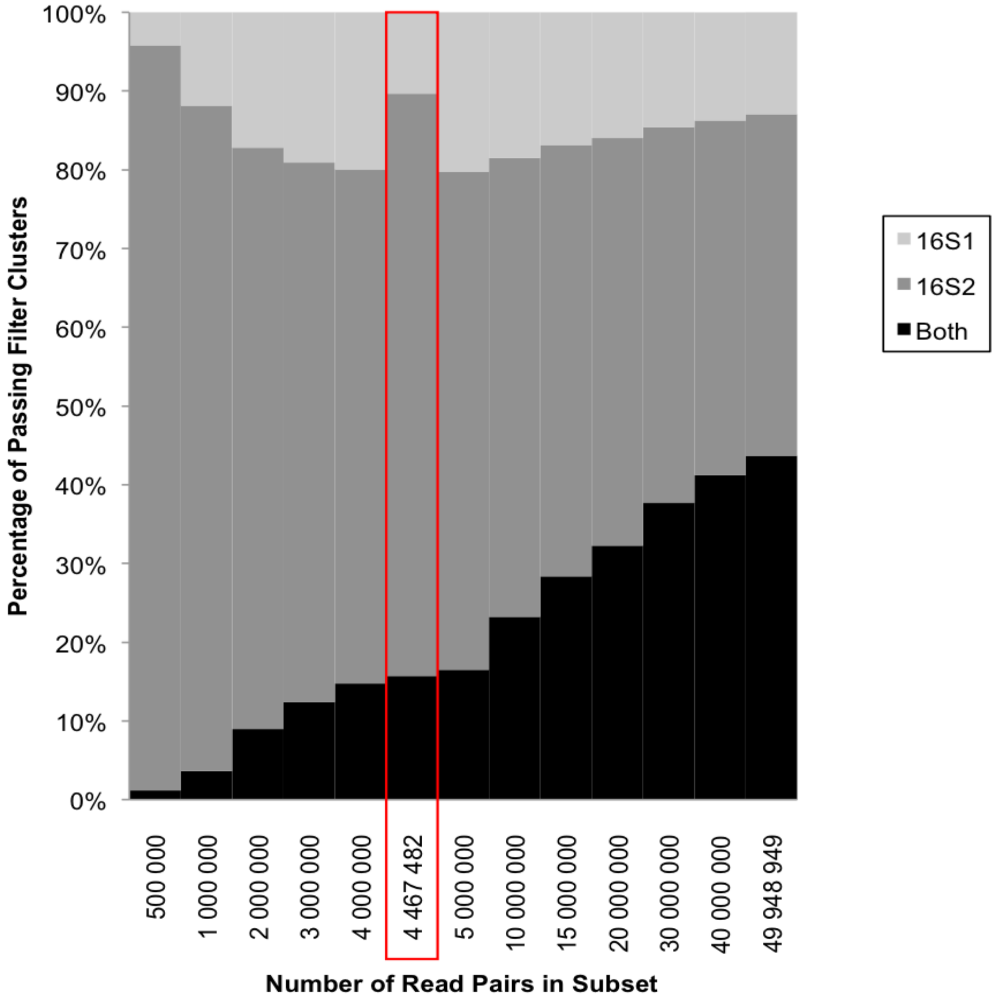

Supplementary Figure 6 | Simulation of sample composition relative to sequencing depth. The figure shows the composition analysis of the Biological Sample with randomized subsets of the sequencing data used as input. Due to filtering steps in the data analysis, clusters featuring both 16S.1 and 16S.2 will be underrepresented when sequenced at low depth. This provides a probable explanation for the seemingly large difference in the composition observed when comparing the Model System with the Biological Sample data (as seen in Figure 2B). The red square shows the composition of the Model System relative to the simulated data and the last point shows the result for the full data set for the Biological Sample.

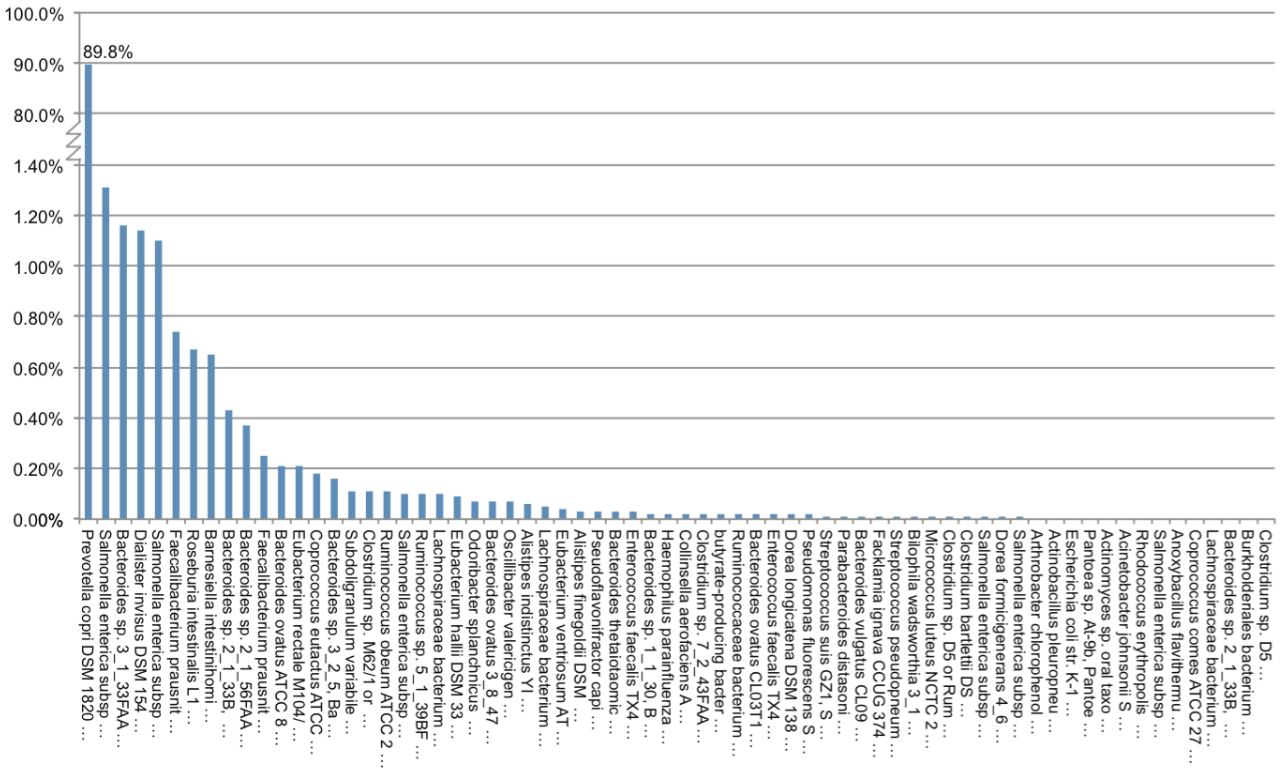

Supplementary Figure 7 | Estimated species distribution in the biological sample. Estimated distribution of known species present in the biological sample. Approximately 90% of the clusters give BLAST hits to *Prevotella copri* DSM 18205. This overrepresentation leads to a high theoretical randomized match rate and any clusters aligning to this species were therefore removed from the analysis to get a more even distribution of bacterial origins. Note the non-continuous scale on the Y-axis.

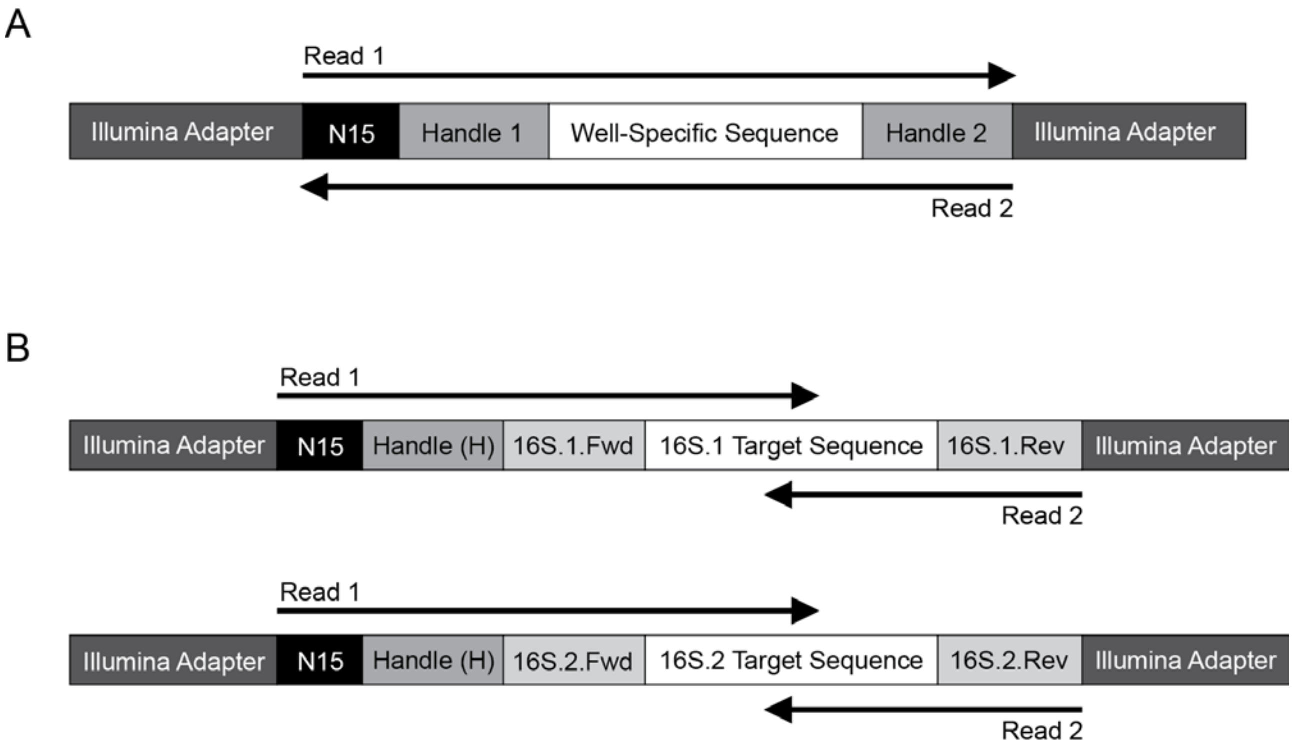

Supplementary Figure 8 | Layout of sequenced library molecules. Schematics of the designed layout of sequenced amplicons for (A) the sorting experiment (validation of monoclonality), and (B) the phasing experiments. Note that the relative length of each element is not in proportion to reality, the target sequences are substantially longer than the sequences at either end.

Supplementary Table 1 | Most common barcode(s) for each sorted well. Distribution of barcode sequences found within each well with successfully amplified products within the five 96 well plates. All barcode sequences with less than 1% representation have been removed. Note that one well were classified as monoclonal even though the most common barcode sequence is represented by less than 90% of the total read population (plate 2 well A1). Also note plate 3 well C5 where there were no “dominant” barcode instead several barcodes were present with an even distribution.

Plate	Well	Count	Sequence	% of well	Monoclonal
2	A1	8 623	ACAGCGACCAGTCCACACAC	84,54%	Yes
2	A11	15 035	GTAACAACTTGGGTGGCACA	99,87%	Yes
2	A4	15 441<br>192	AGATGTGGTAAGGACTAGAG<br>ACAGCGACCAGTCCACACAC	97,80%<br>1,22%	Yes
2	A8	9 724<br>4 305	AACGGAGAAAGCGAGCAACG<br>AAGGCGGAGAAAAAAAAAGA	68,57%<br>30,36%	No
2	B3	10 993	GAAACAAGGGTCGGCTGCGC	99,85%	Yes
2	B4	11 785	GGAGTATGCTGACGAAATAA	99,06%	Yes
2	C1	9 335	TGTCAAAGACATGTTTGGGT	98,93%	Yes
2	C2	9 880	ACAGTAAGTAAGTCAAAAAG	98,97%	Yes
2	C4	13 746<br>304	TGGGTCGACGTAGGGGAAGA<br>GAAACAAGGGTCGGCTGCGC	97,75%<br>2,16%	Yes
2	C6	14 396	AGAGAACCGTAGTAGCATCC	99,83%	Yes
2	D7	14 285	AAGGTCAAGGGCGTGAACAG	99,15%	Yes
2	F11	8 823	GAAAAAAATCGTATAAAATA	99,86%	Yes
2	F5	11 360<br>375	AACACGCGAAGAAGGCACCG<br>AGTCGGAAGTAGGGAAATAT	95,18%<br>3,14%	Yes
2	G12	13 488	ACAAAGAACATTACAGGCGC	99,88%	Yes
2	H12	13 272<br>639	TAACCCATATATAAGGAGAC<br>ACAAAGAACATTACAGGCGC	95,31%<br>4,59%	Yes

			ACTATAAAGC	99,51%	Yes
5	H2	16 461	AAAACCAAAGGAGAATACGC	99,55%	Yes
5	H8	15 308	AAACATGGAAGAGGTGAAGA	99,90%	Yes

Supplementary Table 2A | Primer sequences for sorting experiment

Primer Name	Specification	Primer Sequence 5’ → 3’
Barcoding Oligonucleotide	Used in first emulsion to generate primer library.	CGCCCGCTGCGCTCAGAGAGGATCAAGNNNNNNNNNNNNNNNNNNNNAGATCGGAAGA GCGTCGTGTAGGGAAAGAGTGTAGTCGNCGTCTCTCAAGGCACACAGGGGATAGG
FACS Enrichment	Enrichment of beads with amplified barcodes.	Biotin-CGCCCGCTGCGCTCAGAGAGGATCAAG
FACS Flourescence	Fluorescent labelling of beads for FACS sorting	Cy3–CGCCCGCTGCGCTCAGAGAGGATCAAG
FACS Amplification	Target-specific primer	CGCCCGCTGCGCTCAGAGAGGATCAAG
LnPe 2.0 FACS	Library preparation primer	GTGACTGGAGTTCAGACGTGTGCTCTTCCGATCTAACCAGCACGCGTTATCTTG

Supplementary Table 2B | Primer sequences for phasing experiments

Primer Name	Specification	Primer Sequence 5’ → 3’
Barcoding Oligonucleotide	Used in first emulsion to generate primer library.	AGGAGTGCGGATGGACTTAGNNNNNNNNNNNNNNNAGATCGGAAGAGCGTCGTGTAGG GAAAGAGTGTAGTCGNCGTCTCTCAAGGCACACAGGGGATAGG
Bead Handle	Primer sequence on beads	CCTATCCCCTGTGTGCCTTGAGAGACGNCGACT
emPCR.1.Enrich	Enrichment of beads with amplified barcodes (used after emPCR 1).	Biotin-AGGAGTGCGGATGGACTTAG
16S.1.Fwd	16S.1 Target-specific forward primer (used in emPCR 1)	CTGCTGCCTCCCGTAGGAGTHTAGGAGTGCGGATGGACTTAG
16S.1.Rev	16S.1 Target-specific reverse primer (used in emPCR 2)	GTGACTGGAGTTCAGACGTGTGCTCTTCCGATCTGGACTACCAGGGTATCTAABCCTGT
16S.2.Fwd	16S.2 Target-specific forward primer (used in emPCR 1)	TGTGCGGGYCCCCGTCAATTYMTTTAGGAGTGCGGATGGACTTAG
16S.2.Rev	16S.2 Target-specific reverse primer (used in emPCR 2)	GTGACTGGAGTTCAGACGTGTGCTCTTCCGATCTGAYTTGACGTCRTCCCCDCCTTCCT
16S.1.Enrich	Enrichment of beads with 16S.1 amplicon (used after emPCR 2).	Biotin-THYGTATYACCGCGGCTGCTGGCAC
16S.2.Enrich	Enrichment of beads with 16S.2 amplicon (used after emPCR 2).	Biotin-RCACGAGCTGACGACARCCATGCAVCACCT

Supplementary Note 1 | Detailed data for sorting experiment

Oligonucleotides were designed to, after amplification of FACS sorted beads, produce fragments with the following layout (5' to 3' direction): First an illumina adapter sequence to facilitate cluster generation and sequencing, followed by a stretch of 20 degenerate bases, and a general handle sequence separating the degenerate bases from a identifier sequence (specific for each well in a 96 microtiter plate (previously used by Neiman *et al*.)). This is finally followed by another general handle sequence and the second illumina adapter (Supplementary Figure 8A). The libraries produced in this experiment contains no biological information but instead carries combination of barcodes to be able to asses the number of degenerate barcode oligonucleotides that was compartmentalised with each bead.

Five 96 well plates were prepared as described in Online Methods, whereof 64 wells gave amplicons detectable by capillary gel electrophoresis (wells with a distinct peak for product were chosen for further analysis). Products from these wells were pooled and sequenced and the data were analyzed, as described in Online Methods, producing a histogram of barcodes for each well. Manual evaluation of barcode distributions from the 64 successfully amplified wells reveals that 59 of the wells were monoclonal (predominantly displays one barcode sequence, and this sequence is represented by a major proportion of the read population). Four of the wells displayed two barcodes and the product of one well had an even distribution of a high number of barcodes (see Supplementary Table 1 for barcode distributions of the 64 wells). The results are consistent with what is expected from the Poisson distribution; which gives a theoretical rate between 94.1%-93.2% monoclonal products (Supplementary Figure 3) and 5.65%-6.52% beads with two barcodes at the observed enrichment rate. The representation rate of the most dominant barcode within each monoclonal well ranges from minimum of 84.54% to a maximum of 99.95% of the total barcode population with a mean value of 99.20% and a median value of 99.76%.

The rate of monoclonal amplified products in different emPCR kits is not clearly specified most probably because the non-monoclonal products are filtered during and/or after sequencing. However, the experiments here prove that we are able to produce a population of monoclonally amplified barcoded oligonucleotides attached to the solid surface of a bead.

Supplementary Note 2 | Detailed data for Model System

The sequencing libraries generated from the model system sample were sequenced to a total of 4 467 482 read pairs. In 96.6% of these the handle sequence (“H” in Supplementary Figures 1 and 2) could be identified, confirming that the library molecules originate from the barcoded beads.

78,382 unique 15 base barcode sequences were identified and used to group reads into barcode clusters. 44,701 of these barcode clusters consisted of only one read pair, however as seen in the table below, these clusters only correspond to 1.0% of the total read population. 31,886 of the clusters had only read pairs that either included the illumina adapter sequences or were carrying unexpected primer sequences or combinations. These barcode clusters were excluded from further analysis. After identifying consensus read pairs for the expected amplicons, 43,487 barcode clusters were excluded as the consensus read pairs identified within the barcode clusters did not pass the defined filters (see Supplementary Note 5 for a detailed description of the data analysis). 3,029 barcode clusters had consensus read pairs that passed all filters. 475 of these displayed consensus read pairs corresponding to both the targeted regions, while 313 and 2,241 showed only sequences corresponding to the 16S.1 and 16S.2 target regions, respectively. 91.2% of the total amount of barcode clusters with consensus read pairs passing filter displayed only one version of the target region(s) and were thereby classified as monoclonal. For 411 out of the 418 clusters monoclonal for both target regions, both the consensus read pairs aligned to at least one bacterial subject sequence within the database. For 313 of these clusters the target regions aligned to the same subject sequence, giving a phasing rate of 76.4%.

Table summarising the proportion of original raw reads used in each part of the data analysis.

- Model System -	Number of Read Pairs	% of Read Pairs	Number of clusters
Total Raw Reads	4 467 482	100%	NA
Used for Barcode Clustering	4 088 725	91,5%	78 382
Reads in Single Read Pair Clusters	44 701	1,0%	44 701
Adapter and Primer Dimer Read Pairs	1 519 865	34,0%	NA
Reads in Clusters not Passing Filters	403 756	9,0%	43 487
Reads in Clusters Passing Filters	2 165 104	48,5%	3 029
Reads in Phased Clusters	495 937	11,1%	313

A graphical representation of the number of reads per barcode sequence cluster can be found in Supplementary Figure 5.

Compartmentalization of two genomic fragments from the same species will generate a match even though it is not a true single molecule phasing event. This type of potential false positive phasing events could overestimate the phasing rate. Note that such events are statistically rare given that only around 10% of the droplets are occupied with a genomic fragment and thus the probability of a droplet containing two different fragments from the same species is relatively low. Consequently, the corrected phasing rate presented below is an estimate of the minimum proportion of our data that arise from truly phased events. Still, to enable correction of the observed phasing rate, amplicon sequences were matched randomly between different clusters (as described in Supplementary Note 5d). Note that this random match rate, is based purely on the composition of bacterial species in the sample and is thereby a representation of the hypothetical case when there are always two molecules within each droplet, i.e. it is an estimate of the maximum false positive phasing rate achievable using this dataset. Due to the low complexity of the input sample with only four distinct bacterial 16S sequences, the random match rate is expected to be 25%. The observed rate of 32.0% was larger than this due to an uneven distribution of the four bacterial genomes in the dataset (43.5%, 26.8%, 24.3% and 5.4% of the clusters aligned to *Alteromenas macleodii* strain ATCC 27126, *Paracoccus aminophilus* strain JCM 7686, *Bacillus amyloliquefaciens* strain FZB42 and *Escherichia coli* strain K-12/MG1655, respectively). This skewed distribution results in a higher random match rate ($0.435^2+0.268^2+0.243^2+0.054^2=0.323$) than expected if the four genomes had been equally abundant. The corrected expected match rate of 32.3% more closely resembles the experimentally observed match rate of 32.0%.

The phasing rate was then adjusted to 65.8% using the estimated rate of false positive phasing according to the following calculation scheme:

By definition the total number of barcode sequence clusters (C) is the sum of the clusters stemming from single molecules (S) and the clusters stemming from multiple molecules (M).

(1) $C = M + S$

Furthermore the rate of truly phased single molecule clusters (Ph) is the number of single molecule clusters (S) divided by the total amount (C).

(2) $Ph = \frac{S}{C}$

Likewise the observed rate phased clusters (Ph_{Obs}) was calculated from the number of clusters observed as stemming from single molecules (S_{Obs}) divided by the total amount (C).

(3) $Ph_{Obs} = \frac{S_{Obs}}{C}$

The number of clusters observed as stemming from single molecules (S_{Obs}) are the sum of clusters truly stemming from single molecules (S) and the subpopulation of the clusters that are stemming from multiple molecules (M) that display false positive phasing due to limited library complexity. The size of this subpopulation can be calculated using the rate of false positive phasing (Fp).

(4) $S_{Obs} = S + M * Fp$

Likewise the number of clusters observed to be stemming from multiple DNA molecules (M_{Obs}) can be expressed as any cluster not observed as stemming from a single molecule.

(5) $M_{Obs} = C - S_{Obs}$

The population of clusters truly stemming from multiple DNA molecules (M) are the sum of these observed multi molecule clusters (M_{Obs}) and the earlier mentioned subset of clusters that are falsely observed as stemming from single molecules (M * Fp).

(6) $M = M_{Obs} + M * Fp$

To calculate a corrected value of the phasing rate (Ph) using the observed values and the estimated rate of false positive phasing (Fp_{Est}), the following calculations were made:

In (2) substitute S using (1): $Ph = \frac{(C - M)}{C}$

Solve (6) for M, and substitute M: $M = \frac{M_{Obs}}{1 - Fp_{Est}}$

$$Ph = \frac{C - \frac{M_{Obs}}{1 - Fp_{Est}}}{C}$$

Substitute M_{Obs} using (5): $Ph = \frac{C - \frac{C - S_{Obs}}{1 - Fp_{Est}}}{C}$

Solve (3) for S_{Obs} and substitute S_{Obs}: $S_{Obs} = C * Ph_{Obs}$

$$Ph = \frac{C - \frac{C - C * Ph_{Obs}}{1 - Fp_{Est}}}{C}$$

Simplify: $Ph = 1 - \frac{1 - Ph_{Obs}}{1 - Fp_{Est}}$

Giving a corrected phasing rate of: $Ph = 1 - \frac{1 - 0.7639}{1 - 0.3097} = 65.8\%$

Supplementary Note 3 | Detailed data for Biological System

The sequencing libraries generated from the biological sample were sequenced to a total of 49,948,949 read pairs. In 98.9% of these the handle sequence (“H” in Supplementary Figures 1 and 2) could be identified.

821,491 unique 15 base barcode sequences were identified, out of which 323,066 consisted of more than one read pair. 163,712 were clusters of read pairs with illumina adapter sequences, unexpected primer sequences or combinations and were excluded from the analysis. 591,668 barcode clusters were excluded as none of the consensus read pairs identified within the clusters passed the defined filters. 66,091 barcode clusters had consensus read pairs that passed all filters. 28,889 of these displayed consensus read pairs corresponding to both the targeted regions, while 8,558 and 28,664 showed only sequences corresponding to the 16S.1 and 16S.2 target regions respectively. 91.0% of the total amount of barcode clusters with consensus read pairs passing filter displayed monoclonal amplification of the target region(s). For 21,595 out of 24,361 barcode clusters that that were monoclonal both target regions, both these regions aligned to at least one subject sequence in the database. For 20,210 of these clusters the two target amplicon sequences aligned to the same subject, giving a phasing rate of 93.6%.

Table summarising the proportion of original raw reads used in each part of the data analysis.

- Biological Sample -	Number of Read Pairs	% of Read Pairs	Number of clusters
Total Raw Reads	49 948 949	100%	NA
Used for Barcode Clustering	46 843 835	93,8%	821 491
Reads in Single Read Pair Clusters	498 425	1,0%	498 425
Adapter and Primer Dimer Read Pairs	7 552 726	15,1%	NA
Reads in Clusters not Passing Filters	7 567 822	15,2%	591 668
Reads in Clusters Passing Filters	31 723 287	63,5%	66 091
Reads in Phased Clusters	9 702 631	19,4%	20 210

Note that not all of the 66,091 clusters passing filters could be used for determination of the phasing rate as some had only one of the amplicons or gave no confident BLAST hits towards the database of known bacterial genomes. A graphical representation of the number of reads per barcode sequence cluster can be found in Supplementary Figure 5.

In a highly complex biological sample with thousands of distinct bacterial 16S sequences, the random match rate is expected to be much lower than expected for a mix of four genomes. However, the observed rate of random matching was much greater than the expected (Figure 2C). The distribution of bacteria found in the sample was evaluated and the results show that *Prevotella copri* DSM 18205 constituted 89.8% of the clusters (Supplementary Figure 7). Such an overrepresentation of one type of organism, alone results in an expected random match rate of 80.6% ($0.898^2=0.806$). The corrected expected match rate closely resembles the experimentally observed rate of 75.7%. A more complex sample was simulated by removing sequences aligning to *Prevotella copri* and this resulted in a match rate of 11.9% while the rate of phased barcode clusters remained high at 91.5%.

The phasing rate was then corrected as described in Supplementary Note 2, to 73.1% for the full experiment and 90.3% after removal of *Prevotella copri* DSM 18205.

Supplementary Note 4 | Comparison of Model System and Biological System results

As shown in Figure 2B, the 16S.2 amplicon is generally represented in a higher degree than the 16S.1 amplicon. This uneven amplification is most probably due to differences in amplification efficiency for the different target regions causing a larger proportion of the bead surface to be covered by the shorter 16S.2 amplicon (Supplementary Figure 4). The target-specific primers for the 16S.2 region are also slightly longer, have a higher melting temperature and degree of degeneracy, all these properties may affect the amplification efficiency.

When comparing the results of the model system and the biological sample (see Figure 2B), there is a clear difference in clusters carrying targets from both regions (15.7% and 43.7% respectively). Since the model system was carried out at a smaller scale, it is reasonable to argue that a low sequencing depth will partly contribute to this difference. This is because clusters with enough reads to pass out filters will require a set amount of reads for each amplicon. Clusters where both amplicons are present therefore require twice the amount of reads as single-amplicon clusters (Supplementary Note 5). Note that this cutoff mainly influences the observed proportion of each cluster type, the effect on the phasing rate is negligible. To test this hypothesis we ran a simulation with the Biological Sample data, using only a subset of the sequencing read pairs (Supplementary Figure 6). This simulation confirms that there is a negative relationship between the percentage of clusters with both amplicons and sequencing depth. When the number of sequencing reads is reduced to 4 and 5 million, the percentage of clusters featuring both amplicons falls to 14.7% and 16.5% respectively - very close to the value obtained for the Model System using 4,467,482 reads. It is important to note however, that the amount of sequencing data needed is dependant on size of the library or the amount of beads produced in each assay.

The difference in reduction of average hit list lengths in Figure 2D between the Model System and the Biological Sample is due to sample complexity and 16S sequence composition. The large difference observed between the Biological Sample with and without the most abundant species is explained by the fact that this species only aligned to one subject sequence when classified.

Supplementary Note 5 | Data analysis for phasing experiments

a. Identification and clustering of barcode sequences

Initially the start and end positions of the handle sequence was identified within the first read. This was done by finding the subsequence with minimal hamming distance to the known handle sequence. The barcode sequence was then defined as the stretch of bases between the read start and the start position of the handle (Supplementary Figure 8B). A maximum of 4 mismatches was allowed in the handle sequence. The number of mismatches detected was evaluated to estimate the substitution error rate in this part of the read that is adjacent to the barcode sequence. This estimate does not consider any other types of error than substitution and if more than four mismatches are present in the the handle the read will not contribute to the statistics. However, this is not valid for the full sequence as the error rate varies along the reads. This estimate was therefore used solely as a guideline for determining the maximum number of mismatches in the barcode clustering. In the Model system and Biological sample this substitution error rate was determined to 0.25% and 0.22% respectively, which theoretically should have a minor effect on the clustering of barcode sequences. Barcode sequences were clustered using DNAclust[3] allowing for a predefined number of non-identical positions and with the 100 most abundant unique sequences as "predetermined cluster centers".

To determine the most appropriate identity cutoff to use in the clustering, the rate of barcode sequence clusters carrying monoclonally amplified target regions was evaluated for the Model System data with increasing number of non-identical positions. For 0, 1, 2 and 3 non-identical positions the rate of clusters with monoclonal amplifications were 92.1%, 91.9%, 90.5% and 77.3%, respectively. No more than one mismatch should theoretically be present in the 15 base barcode sequence, considering the error rate estimated during the handle sequence identification, meaning that allowing more than one non-identical position is without effect for correct clustering of barcodes. In addition, the risk of two 15 bases random barcodes to be within an edit distance of one is small as the model system is based on a subset of only 10^5 barcode sequences (barcoded beads) out of 4^15 possible sequences. The clustering should therefore be considered as correct, though the decreased monoclonality indicates that reads with substitutions in the barcode sequence are prone to carry other kinds of errors in connection to the target amplicon sequences for example chimeric sequences formed during sequencing library amplification (see section c.iii below). To further investigate this, simulated datasets was constructed with varying rate of mismatches (0.0%, 0.1% and 0.5%) with a total of 1000 unique barcode sequences per dataset and between 100-1000 read pairs per unique barcode sequence (randomly selected for each barcode). Results indicate that an error rate of only 0.1% has a large effect on the total number of barcode sequence clusters identified (data not shown). Furthermore, false clustering is often observed when trying to cluster barcode sequences containing homopolymeric stretches. Using another method (for the initial clustering procedure) might improve the results significantly. Considering this, to guarantee correct grouping of read pairs, clustering was based on unique barcode sequences without allowing for non-identical positions in all analyses. To avoid large amounts of clusters resulting from sequencing and amplification errors in the barcode sequences, read count cutoffs were used (see section c for further details).

b. Filtering of reads

Reads containing illumina adapter sequence or not having a correct combination of primer sequences were removed. Illumina adapter content were identified by searching the read sequences for substrings matching the first 20 bases of the adapter with a hamming distance less than three. Primer sequences were identified by matching read sequences and the known target-specific primer sequences without allowing mismatches.

c. Clustering of reads with target-specific sequence content

For read pairs remaining after filtering, the barcode, handle and primer sequences were removed. The two single reads were concatenated to one continuous sequence with a separator sequence (N_{10}). Clustering of these sequences was then performed within each barcode sequence cluster using CD-HIT-454[4]. The standard identity cutoff of 97% was used to cluster similar sequences. The two individual single reads were reconstructed after clustering by splitting the consensus using the separator sequence, creating consensus read pairs for the targeted amplicons. Consensus read pairs not fulfilling all of the following criteria were filtered; (c.i) The consensus read pair was based on an absolute count of at least 30 original read pairs. (c.ii) The consensus read pair was supported by at least 10% of the total number of reads mapping to the same unique barcode sequence. (c.iii) If more than one consensus read pair was present for a target region, any such sequence had to be supported by at least 40% of the number of read pairs that supported the most common consensus read pair. Each condition is explained and motivated below.

c.i. To exclude barcode sequence clusters resulting from sequencing and amplification errors, 30 read pairs was required for each consensus read pair to pass filters. Simulated data with 0.1% mismatches was used to determine the number of reads supporting each consensus read pair required to find the expected number of barcode clusters. When 50, 100 or 250 read pairs were generated for each barcode sequence cluster, two pairs for each consensus were enough to obtain the expected clusters. For 500 and

1000 read pairs per barcode cluster the suitable cutoff was determined to five reads (data not shown), indicating that more pairs are required if the average number of reads per barcode cluster is higher. To account for a higher error rate and improve the quality of our consensus sequences we chose the commonly used read cutoff of 30 reads.

c.ii. Furthermore, to account for the trend observed for the barcode cluster generated with high number of read pairs, a cutoff of 10% of the total number of reads mapping to the specific amplicon type was required for each consensus sequence to pass filters.

c.iii According to the Poisson distribution only one or two (in rare cases three or more) read pair consensus sequences are expected per targeted region. For some barcode sequence cluster many more are observed, contradicting the results from the sorting experiment. A large proportion of these consensus read pairs are often supported by only a minor part of reads present within the barcode sequence cluster indicating that a low level leakage between barcode clusters might occur. One plausible explanation for this is formation of chimeric sequences during library amplification. For such an event the barcode sequence from one compartment (and possibly part of the target amplicon sequence) might be connected to (a part of) the target amplicon sequence from another compartment. To remove background from possible chimeric sequences all read pair consensus sequences were filtered based on the assumption that two molecules from the same target region will have similar sequence content and therefore amplify evenly. A criterion was introduced that require any consensus read pair to be based on at least 40% the number of reads of the most represented consensus (for the same target region). A 60% variation in abundance is thereby allowed for variants of an amplicon sequences, any consensus read pairs not fitting this model were removed.

Finally, after filtering of consensus read pairs, each barcode sequence cluster was classified as either monoclonal or polyclonal. The classification was based on the number of consensus read pairs for each of the target regions, one or zero consensus read pairs per target were classified as monoclonal while targets displaying more than one consensus read pair were classified as stemming from several separate DNA fragments.

d. Classification of target amplicon consensus sequences.

The rate of true single molecule phasing was investigated to determine the proportion of barcode sequence clusters with monoclonally amplified target regions that stemmed from one single DNA molecule as opposed to a scenario where the two regions stemmed from two separate molecules. This was done by determining potential bacterial origins of the consensus read pair sequences by aligning the sequences to a database of bacterial genomes (concatenation of ftp://ftp.ncbi.nlm.nih.gov/genomes/HUMAN_MICROBIOM/Bacteria/all.fna.tar.gz and ftp://ftp.ncbi.nlm.nih.gov/genomes/Bacteria/all.fna.tar.gz both downloaded 2013-08-12) using BLAST+[5]. For each consensus read pair a hit list was constructed containing the organisms that both reads aligned to with at least 97% identity and 95% alignment length coverage. The hit lists of the two target regions were compared and any organism present in both were recorded creating an overlap list for each barcode cluster where both the target regions displayed monoclonal amplification and had at least one alignment passing the defined cutoffs. All barcode clusters where an overlap between the hit lists for the two targeted amplicons was observer was considered successfully phased. False positive phasing may occur due to matches by stochastic compartmentalization of two single DNA molecules with identical bacterial origin. To estimate this rate the population of hit lists were shuffled and one hit list from each targeted region were picked randomly and compared. This estimate was considered as the upper limit of the phasing rate that can be explained by false positive phasing for the given bacterial distribution. The distribution of bacteria found within the sample was evaluated by only considering barcode sequence clusters with monoclonal amplification of both the targeted regions and where the bacterial origin of those regions overlapped and then the number of occurrences for each unique overlap list. For the biological sample one bacterial species was highly abundant in the sample resulting in that a large proportion of the phasing could be explained by false positives (Supplementary Note 3). Sequences aligning to the most highly abundant species were therefore removed from the dataset and thus considerably reducing the false positive phasing while the match rate remained unchanged. The alignment data was also used to evaluate the potential utility of the method in a biological study by investigating the effect of phasing information on bacterial identification. This was done by counting the number of alignments hits for each of the two target amplicons as well as the number of hits present for both regions (the overlap), for all clusters where the lists of alignment hits overlapped at least once. The average number of potential bacterial origins were then calculated for each the two target regions separately as well as for the combined information (the overlap). For this evaluation the Model System were aligned to the full database.

Supplementary Note 6 | Phasing of pre-amplified sequences

We investigated the feasibility of applying this method to analyze DNA sequences preamplified by long range PCR rather than extracted whole genomic material. Primers were used to amplify the entire 16S rRNA gene for each of the Model system genomes. The PCR products were then purified and an equimolar pool was prepared and diluted before adding it together with barcoded beads in the second emulsion reaction. Apart from the input material the sample was processed precisely as the Model System and the Biological Sample (Online Methods).

The sample was sequenced at a similar depth as the Model System, generating 4,212,164 read pairs used for clustering. 3,478 unique clusters were identified with consensus read pairs passing all filters. 88.5% of these clusters displayed monoclonal amplification of the targets regions(s). 10.6% and 64.5% of the barcode clusters had amplified 16S.1 and 16S.2 respectively, while both amplicons could be identified for 24.9% of the clusters. Sequence phasing was determined as successful for 84.6% of the barcode clusters, and the random match rate was 25.8% - close to the theoretical 25% expected from an even mixture of four genomes. These results indicate that our method could be applied to any targeted analysis of genomic material, such as the amplification and phasing of the ~5 kb HLA-A gene.

Supplementary References

1. Cole, J.R. *et al.* Ribosomal Database Project: data and tools for high throughput rRNA analysis. *Nucleic Acids Res.* **42** D633-642 (2014)

2. Hugerth, L.W. *et al.* DegePrime, a Program for Degenerate Primer Design for Broad-Taxonomic-Range PCR in Microbial Ecology Studies. *Appl. Environ. Microbiol.* **80** 5116-5123 (2014)

3. Ghodsi, M., Liu, B. & Pop, M. DNACLUST: accurate and efficient clustering of phylogenetic marker genes**.** *BMC Bioinformatics* **12** 271 (2011)

4. Niu, B., Fu, L., Sun, S. & Li, W. Artificial and natural duplicates in pyrosequencing reads of metagenomic data. *BMC Bioinformatics* **11** 187 (2010)

5. Camacho, C. *et al*. BLAST+: architecture and applications. *BMC Bioinformatics* **10** 421 (2009)
